# Illness Perception Accorded by Language Assistance in Non-Japanese-Speaking Patients

**DOI:** 10.7759/cureus.50532

**Published:** 2023-12-14

**Authors:** Yue Wang, Akira Oonishi, Anna Kazami, Ruriko Suminaga, Enari Den, Zhuo Li, Naoko Ono, Francois Niyonsaba, Ai Ikeda

**Affiliations:** 1 Department of Medical Interpreting, Graduate School of Medicine, Juntendo University, Tokyo, JPN; 2 Faculty of International Liberal Arts, Juntendo University, Tokyo, JPN

**Keywords:** healthcare, health communication, non-japanese-speaking patients, illness perception, language assistance

## Abstract

Background and objective

While over half of foreign residents in Japan need language assistance during medical consultations, no extant studies have clarified illness perception accorded by language assistance in non-Japanese-speaking patients. This cross-sectional study conducted an online questionnaire survey to investigate the illness perception of non-Japanese-speaking patients and analyze the factors related to illness perception.

Methodology

The survey was conducted twice, from February to May 2022 and from February to April 2023, targeting non-Japanese-speaking individuals. In total, 293 valid responses were obtained. The Brief Illness Perception Questionnaire (Brief IPQ) scores were compared between the groups receiving language assistance and those without assistance, and a logistic regression analysis was performed to examine the factors related to illness perception accorded by the status of the language assistance group.

Results

The total score for illness perception was significantly lower in the language assistance group than in the non-assistance group (*P* = 0.04). Moreover, in the language assistance group, age (odds ratio [OR] = 0.91, 95% confidence interval [CI] = 0.84-0.99) and comprehension of medical consultations (OR = 0.31, 95% CI = 0.11-0.83) were significantly associated with low illness perception among participants. However, these associations were not observed in the non-assistance group.

Conclusions

These findings underscore the crucial role of ensuring effective communication and promoting a better understanding of illness perception during medical consultations.

## Introduction

Japan is currently grappling with a declining birth rate and an aging population, which have reduced the labor force [1]. In response to the labor shortage, the Japanese government revised the Immigration Control and Refugee Recognition Act in 2018 to facilitate the acceptance of foreign workers and improve their workplace and social conditions [2]. The government estimated that approximately 345,000 workers would be accepted over five years starting in April 2019 [3]. Moreover, according to Tokyo-based public think tanks, Japan will need 6.74 million foreign workers by 2040. The influx of foreign residents has significantly influenced various aspects of Japanese society, including the healthcare system [4].

The Basic Survey on Foreign Residents in FY2022 [5] revealed that over half of foreign residents (52.8%) required language assistance when seeking medical care. When visiting a hospital, 27.2% of foreign residents consulted a family member, relative, or friend who spoke Japanese (ad hoc interpreters); 10.6% used a multilingual translator or application (artificial intelligence [AI] translator); and 2.2% requested a medical interpreter. Given that patients’ perceptions are developed based on the information they receive from formal and informal sources, including healthcare providers, media, family, friends, and fellow patients [6], it is reasonable to assume that language assistance may significantly impact non-Japanese-speaking patients’ illness perception.

In the realm of healthcare, the concept of *illness perception* holds significance. Illness perception refers to “the representations and beliefs that people have about their symptoms and illnesses” [7-9]. Numerous studies [10-13] and meta-analyses [14,15] have demonstrated that illness perception influences patient outcomes, such as treatment adherence and quality of life. However, most studies of illness perception have been conducted among native speakers with specific diseases. Although some studies have focused on specific ethnic minority groups with diabetes mellitus [16,17], they typically overlooked the influence of language assistance on non-native-speaking patients. Considering that approximately half of foreign residents in Japanese society require language assistance when seeking medical care, it is imperative to delve into patients’ illness perceptions, encompassing both those receiving language assistance and those who are not.

Therefore, this study aimed to examine illness perception accorded by language assistance in non-Japanese-speaking patients through a questionnaire survey and analyze related factors. The present results are expected to promote behavioral changes among healthcare providers and improve access to healthcare in communities.

## Materials and methods

Setting and participants

An online questionnaire survey was conducted twice, from February to May 2022 and from February to April 2023, targeting non-Japanese-speaking individuals aged 18 years or older who sought medical care at hospitals in Japan. This study was approved by the Research Ethics Committee of the Faculty of Medicine of Juntendo University (approval numbers: E21-0237-M02 and E21-0237-M04). 

**Figure 1:**
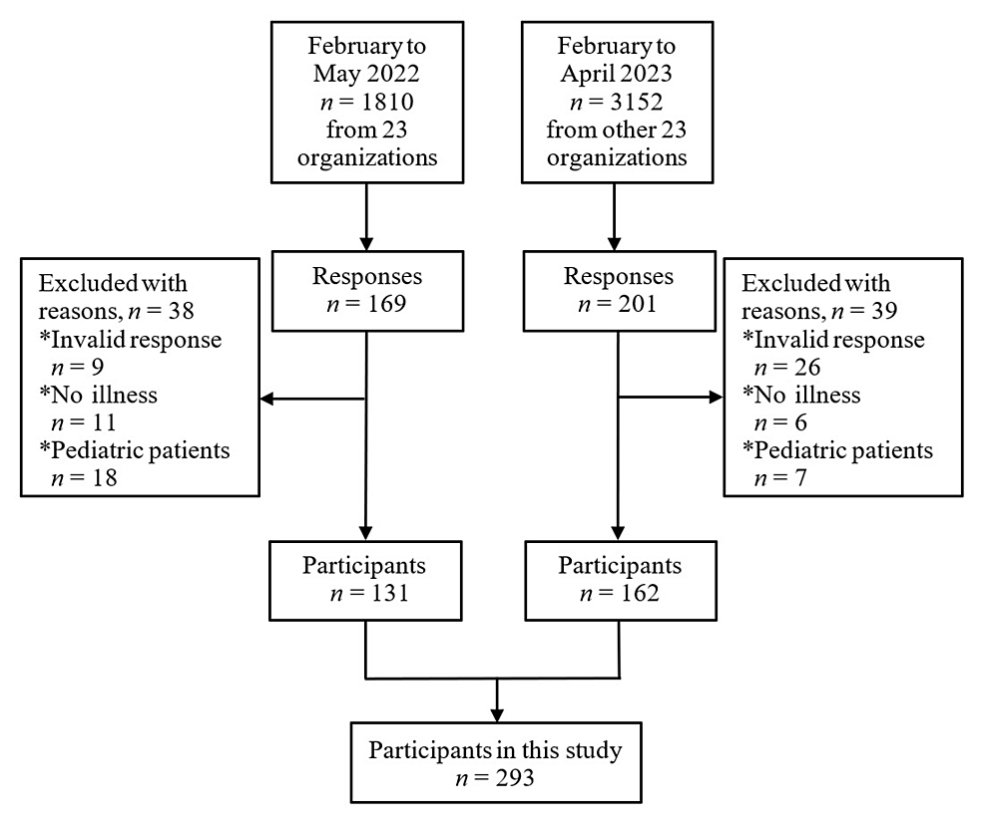
Flowchart of the participant’s selection process. Image credit: Yue Wang.

The questionnaire was available in multiple languages, including Japanese (phonograms added to Chinese characters), English, Chinese, and Vietnamese, which allowed participants to choose the most suitable language for their responses. A total of 46 organizations, including Japanese language support networks, cooperative leagues of commerce and industry, and international associations, volunteered to participate in this study and distributed an online questionnaire to 4,962 non-Japanese-speaking residents who were members of these organizations. Figure 1 presents a flowchart of the participant selection process. The survey was conducted from February to May 2022, and 169 responses were collected from 23 organizations with a response rate of 9.4% (*n* = 169/1,810). In a subsequent survey conducted between February and April 2023, 201 responses were collected from other 23 organizations, resulting in a response rate of 6.4% (*n* = 201/3,152). After excluding invalid responses, the final sample size was 293 participants. Although the response rate of the study is below 10%, it is reasonable for internet-based surveys [18].

Measures

The anonymous online questionnaire used in this study comprised three main parts. Part 1 collected basic background information using the following six items: age, sex, nationality or region, residence status, duration of living in Japan, and language used in daily life. Part 2 focused on participants’ medical care experiences at hospitals in Japan regarding their current or previous treatments. Three items from Part 2 were used in this study. Item 1 asked participants to indicate their method of communication when interacting with physicians, using options such as direct conversations, AI translators, or an interpreter. Participants who conversed directly with physicians were categorized into the non-assistance group, whereas those who used AI translators or interpreters were categorized into the language assistance group. Item 2 measured the participants’ self-reported degree of comprehension of conversations with the physician using a scale from 1 to 4. A score of 1 represented the worst possible comprehension, and a score of 4 represented the best possible comprehension. Participants who scored 1 or 2 were categorized as having no comprehension of medical consultations, whereas those who scored 3 or 4 were categorized as having comprehension. Item 3 was open-ended, asking participants to name their disease. Part 3 included the Brief Illness Perception Questionnaire (Brief IPQ) [19]. The illness perception of the participants was assessed using the Brief IPQ, which comprises nine items representing the nine dimensions of the construct. The first eight items, (1) consequences, (2) timelines, (3) personal control, (4) treatment control, (5) identity, (6) concern, (7) coherence, and (8) emotional response, were assessed using a Likert scale from 0 to 10. The last item (9) was open-ended and asked patients to indicate the three most important causes of their disease. The scores on Items 3, 4, and 7 were reversed such that, for each item, higher scores indicated stronger perceptions. The total score ranges from 0 to 80, with higher scores indicating higher threat perceptions. The Brief IPQ cutoff point for interpreting the level of threat perception (low or high) was determined based on the median of the total score (32) in this study. A score ≥32 was used to define the category of high threat perception, and a score <32 was used to define the category of low threat perception.

Statistical analysis

Data were analyzed using IBM SPSS Statistics for Windows, Version 29 (IBM Corp., Armonk, NY). The significance level was set at *P* < 0.05 with a two-tailed test. Chi-square and Mann-Whitney U tests were used to compare background information, the degree of comprehension of the consultation conversation, the scores of each item of the Brief IPQ, and the total score of the Brief IPQ between the language assistance and non-assistance groups. Subsequently, a logistic regression analysis was performed to examine the relationships between basic background characteristics, comprehension of medical consultations, and illness perception accorded by the language assistance and non-assistance groups.

## Results

Table 1 lists the participants’ background information. The mean age of the participants was 31.25 years (standard deviation [SD] = 11.19, range 18-76), with a median age of 27 years (interquartile range [IQR] 23.50-37.00). Of the total number of participants, 129 (44%) were men and 164 (56%) were women. The majority of the participants were from China (115/293, 39.2%), followed by Vietnam (107/293, 36.5%). In terms of residence status, the three primary categories were students (86/293, 29.4%), technical intern training (70/293, 23.9%), and status-based (46/293, 15.7%, encompassing permanent residents, special permanent residents, spouses or children of Japanese nationals, and spouses or children of permanent residents). Of the 293 participants, approximately 225 (64.8%) had resided in Japan for one to five years and 181 (61.8%) reported using Japanese daily.

**Table 1:** Descriptive characteristics of various populations by language assistance. ^a^The Mann-Whitney U test was used to compare age between the language assistance and non-assistance groups, whereas the chi-square test was used for other variables. ^b^*Specialized or technical fields* include highly skilled foreign professionals, attorneys, judicial scriveners, public accountants, tax accountants, artists, etc. ^c^*Specified skilled worker* includes specified skilled workers (work-ready foreign nationals who possess certain expertise and skills in certain industrial fields). ^d^*Based on status* includes permanent residents, special permanent residents, spouse or child of a Japanese national, or spouse or child of a permanent resident. ^e^*Technical intern training* includes trainees (technical interns) in a local government. SD, standard deviation

	Whole samples		Language assistance				*P*-value
	*n* = 293		Yes (*n* = 101)		No (*n* = 192)		
	n	%	n	%	n	%	
Age ^a^							0.11
Mean ± SD	31.3 ± 11.2		30.0 ± 10.8		31.9 ± 11.4		
Median	27		26		28		
IQR	23.5-37.0		23-34		24-38		
Sex							0.72
Male	129	44	43	42.6	86	44.8	
Female	164	56	58	57.4	106	55.2	
Nationality/region							<0.001
China	115	39.2	27	26.7	88	45.8	
Vietnam	107	36.5	54	53.5	53	27.6	
Other Asian country	41	14	12	11.9	29	15.1	
North America and Europe	15	5.1	4	4	11	5.7	
Latin America and Africa	15	5.1	4	4	11	5.7	
Residence status							<0.001
Specialized or technical fields^b^	45	15.4	10	9.9	35	18.2	
Specified skilled worker^c^	19	6.5	5	5	14	7.3	
Based on status^d^	46	15.7	15	14.9	31	16.1	
Technical intern training^e^	70	23.9	44	43.6	26	13.5	
Students	86	29.4	15	14.9	71	37	
Others	27	9.2	12	11.9	15	7.8	
Duration of living in Japan							0.003
<1 year	35	11.9	20	19.8	15	7.8	
1-5 years	190	64.8	67	66.3	123	64.1	
6-10 years	42	14.3	11	10.9	31	16.1	
11-20 years	10	3.4	2	2	8	4.2	
≥21 years	16	5.5	1	1	15	7.8	
Using Japanese in daily life							<0.001
No	112	38.2	53	52.5	59	30.7	
Yes	181	61.8	48	47.5	133	69.3	

In this survey, 192 (65.5%) participants conversed directly with the physician, while 101 (34.5%) used various communication aids, including ad hoc interpreters (53, 52.5%), AI translators (29, 28.7%), nonmedical interpreters (13, 12.9%), and medical interpreters (6, 5.9%). A chi-square test revealed significant relationships between language assistance and several variables, including *nationality or region*, *residence status*, and *using Japanese in daily life*, with *P* < 0.001, as well as *duration of living in Japan* (*P* = 0.003).

Table 2 shows the Brief IPQ scores and the degree of comprehension of the consultation conversation between the language assistance and non-assistance groups. The comprehension of consultation conversations was significantly lower in the language assistance group than in the non-assistance group (*P* < 0.001). Regarding the Brief IPQ subscales, the language assistance group had lower scores for *timelines* (*P* < 0.001), *identity* (*P* < 0.001), and *emotional response* (*P* = 0.02). Although the language assistance group had a higher score for *coherence* (*P* = 0.04), this indicates lower levels of understanding due to the reversed nature of the *coherence* item. The total Brief IPQ score was significantly lower in the language assistance group than in the non-assistance group (*P* = 0.04).

**Table 2:** Scores of Brief IPQ and the degree of comprehension of consultation conversation. ^a^*Personal control*, *treatment control*, and *coherence* are reversed items. Brief IPQ, Brief Illness Perception Questionnaire; IQR, interquartile range

	Language assistance				
	Yes (*n* = 101)		No (*n* = 192)		*P* for difference
	Median (IQR)	Mean rank	Median (IQR)	Mean rank	
Degree of comprehension	2.0 (2.0-3.0)	99.1	3.0 (3.0-4.0)	172.3	<0.001
Brief IPQ item					
Consequences	2.0 (0.5-6.0)	140.7	3.00 (1.0-6.0)	150.3	0.36
Timelines	1.0 (0.0-3.0)	135.4	3.0 (1.0-7.0)	164.6	<0.001
Personal control^a^	4.0 (0.0-8.0)	150.4	4.0 (2.0-6.0)	145.2	0.61
Treatment control^a^	2.0 (0.0-5.0)	140.1	3.0 (0.0-6.0)	150.7	0.30
Identity	2.0 (0.0-5.0)	121.1	4.0 (1.3-6.0)	160.7	<0.001
Concern	4.0 (0.0-8.0)	134.1	5.0 (3.0-8.0)	153.8	0.06
Coherence^a^	4.0 (0.0-7.0)	160.6	2.0 (1.0-5.0)	139.8	0.04
Emotional response	3.0 (0.0-5.5)	131.4	5.0 (1.0-7.0)	155.2	0.02
Total score	29.0 (15.0-40.0)	133.3	33.0 (24.0-41.0)	154.2	0.04

Table 3 shows the univariate and multivariate regression estimates of the odds ratio (OR) for low threat perception among participants in the language assistance group. In the univariate analysis, age was found to be significantly associated with low threat perception (OR = 0.93, 95% CI = 0.89-0.98). Female participants had higher odds of lower threat perception (OR = 0.44, 95% CI = 0.19-0.98) than male participants; Vietnamese participants showed significantly higher odds of outcome (OR = 6.18, 95% CI = 2.23-17.1) compared to Chinese participants; and participants who comprehended the context of medical consultations had lower threat perception (OR = 0.34, 95% CI = 0.15-0.77). In the multivariate analysis, age (OR = 0.93, 95% CI = 0.84-0.99) and participants who comprehended the context of medical consultations (OR = 0.31, 95% CI = 0.11-0.83) also had lower threat perception. However, sex and nationality/region were not significantly associated with outcomes. 

**Table 3:** Univariate and multivariate regression estimates of odds ratio for low illness perception outcome among participants in the language assistance group (n = 101). ^a^Multivariate regression via the forced entry procedure was used to analyze the data. ^*^P < 0.05. ^**^P < 0.001.

	Univariate		Multivariate^a^	
	Odds ratio	95% CI	Odds ratio	95% CI
Age	0.93	0.89-0.98*	0.93	0.84-0.99*
Sex				
Male	1.00 (reference)		1.00 (reference)	
Female	0.44	0.19-0.98*	0.43	0.16-1.17
Nationality/region				
China	1.00 (reference)		1.00 (reference)	
Vietnam	6.18	2.23-17.1**	5.43	0.91-32.3
Other Asian countries	1.70	0.41-6.98	1.68	0.31-9.13
Rest of the world	0.79	0.13-4.79	1.29	0.13-11.4
Residence status				
Specialized or technical fields	1.00 (reference)		1.00 (reference)	
Training and skilled workers	3.09	0.76-12.5	0.64	0.10-4.55
Based on status	0.75	0.14-3.94	1.73	0.19-15.8
Students	1.31	0.26-6.64	0.80	0.08-7.06
Others	1.07	0.19-5.91	2.04	0.20-21.2
Duration of living in Japan				
<6 years	1.00 (reference)		1.00 (reference)	
≥6 years	0.85	0.28-2.63	5.91	0.76-45.9
Using Japanese in daily life				
No	1.00 (reference)		1.00 (reference)	
Yes	2.01	0.91–4.47	1.75	0.61–5.06
Comprehension of medical consultations				
No	1.00 (reference)		1.00 (reference)	
Yes	0.34	0.15-0.77*	0.31	0.11-0.83*

Notably, when the same univariate and multivariate regression analyses were performed among participants in the non-assistance group, no significant associations with lower threat perception were observed (Table 4).

**Table 4:** Univariate and multivariate regression estimates of odds ratio for low illness perception outcome among participants in the non-assistance group (n = 192). ^a^Multivariate regression via the forced entry procedure was used to analyze the data.

	Univariate		Multivariate ^a^	
	Odds ratio	95% CI	Odds ratio	95% CI
Age	0.99	0.96-1.01	0.98	0.95-1.02
Sex				
Male	1.00 (reference)		1.00 (reference)	
Female	1.02	0.58-1.80	1.01	0.54-1.88
Nationality/region				
China	1.00 (reference)		1.00 (reference)	
Vietnam	1.74	0.87-3.49	1.59	0.62-4.10
Other Asian countries	0.96	0.41-2.22	1.24	0.47-3.29
Rest of the world	1.43	0.56-3.65	2.47	0.76-7.98
Residence status				
Specialized or technical fields	1.00 (reference)		1.00 (reference)	
Training and skilled workers	2.48	0.97-6.36	2.31	0.71-7.53
Based on status	1.38	0.51-3.76	1.72	0.57-5.34
Students	1.58	0.69-3.65	1.65	0.61-4.48
Others	2.19	0.64-7.50	2.40	0.68-8.50
Duration of living in Japan				
<6 years	1.00 (reference)		1.00 (reference)	
≥6 years	0.80	0.42-1.51	0.87	0.38-1.99
Using Japanese in daily life				
No	1.00 (reference)		1.00 (reference)	
Yes	1.21	0.65–2.24	1.33	0.68–2.63
Comprehension of medical consultations				
No	1.00 (reference)		1.00 (reference)	
Yes	1.08	0.54-2.13	1.40	0.66-2.97

## Discussion

To our knowledge, this study is the first to investigate illness perception in non-Japanese-speaking patients accorded by language assistance. A significant finding was that patients in the language assistance group exhibited lower comprehension of consultation conversations, less understanding of their diseases, and lower levels of illness perception than those in the non-assistance group. Additionally, within the language assistance group, specific factors such as age and ability to understand medical consultations were related to illness perception.

By comparing the Basic Survey of Foreign Residents in FY2022, reported by the Japanese government [5], this study found similarities and differences in participants’ background characteristics. Both sources show that approximately half of foreign residents had resided in Japan for less than 10 years, with China and Vietnam being the two main countries of origin. However, there is a disparity in the distribution of residential status between these two sources. The government report revealed *permanent residents* (30.4%); *engineers, specialists in humanities*, and *international services* (14.4%); and *students* (10.7%) as the top categories. In this study, most participants were categorized as *students* (29.4%); *technical intern trainees* (23.9%); and *engineers, specialists in humanities, and international services* (15.4%). Consequently, the participants in this study were relatively young (mean age ± SD = 31.3 ± 11.2) and had a shorter overall life experience in Japan. Considering the increasing number of young foreign workers in Japan, the unique characteristics of the study participants provide valuable insights for future research.

Approximately one-third of the participants did not know the name of their disease. Another third provided information on various medical conditions, whereas the remaining participants listed more than 20 different disease names. Notably, no serious diseases, such as malignant tumors or cardiovascular disease, were mentioned. Medical conditions primarily included common issues such as colds, allergies, and injuries. The participants in the language assistance group commonly used ad hoc interpreters and AI translators. However, the utilization of medical interpreters was rare, which is consistent with the findings of a Japanese government report [5].

In this context, the study’s data showed that illness perception among participants with these low-risk conditions differed between the language assistance and non-assistance groups. The language assistance group reported weaker appreciation of their diseases (timelines), fewer somatic complaints (identity), and poorer understanding (coherence) than the non-assistance group. These results suggest that ad hoc interpreters or AI translators while providing valuable forms of language assistance may not be sufficient for effective communication and for promoting a better understanding of illness perception during medical consultations. This is because they may not possess the expertise of medical interpreters to convey complex medical information accurately or maintain cultural sensitivity.

Previous research [20,21] has consistently demonstrated that medical interpreters are associated with better clinical care than ad hoc interpreters. Relying solely on ad hoc interpreters or AI translators may pose risks to patients and healthcare providers in terms of treatment adherence and overall healthcare outcomes. Considering the outcomes for non-Japanese-speaking patients, healthcare providers and government agencies should improve the availability of medical interpreters to maximize effective communication and provide better clinical care for diverse linguistic populations.

Additionally, younger participants and those with a lower comprehension of medical consultations exhibited lower threat perception in the language assistance group. In contrast, no associations between background characteristics and illness perception were observed among participants in the non-assistance group. These findings suggest that a one-size-fits-all communication approach may not be effective for non-Japanese-speaking patients. Given these results, physicians should consider tailoring their care approaches based on the patient’s background characteristics to match the patient’s comprehension. While communication skills were not the primary focus of this study, the vigorous promotion of *Easy Japanese* at medical institutions is necessary. *Easy Japanese*, a simple, easy-to-understand way of speaking Japanese, as proposed by Sato [22], can enhance communication between healthcare providers and patients in medical settings. Medical professionals have successfully implemented *Easy Japanese* in medical education [23]. Furthermore, health literacy programs can assist patients in better understanding medical terms, procedures, and the nature of their illnesses [24]. This consideration is crucial for ensuring effective communication and promoting a better understanding of illnesses, particularly when developing strategies aimed at enhancing healthcare access and outcomes for non-Japanese-speaking patients.

This study provided important findings regarding illness perception accorded by language assistance. However, this study had several limitations. First, participants with serious illnesses were not included. Future research should consider this group to better understand variations in illness perceptions. Second, this study used a cross-sectional design that retrospectively asked participants about their clinical experiences. This approach may introduce bias in illness perception as it can change over time. Finally, owing to the small sample size of the language assistance group, this study did not discuss the effects of various language aids on illness perception. Studies with larger sample sizes and a longitudinal design are required to address these limitations.

## Conclusions

To the best of our knowledge, this is the first study to clarify illness perception in non-Japanese-speaking patients accorded by language assistance. The findings demonstrated significant differences in illness perception between the language assistance and non-assistance groups. Additionally, age and comprehension of medical consultations were significantly associated with low threat perception among participants in the language-assistance group, whereas no significant association was observed in the non-assistance group. These findings suggest that methods of language assistance and effective communication should be carefully considered in medical settings. Although future surveys should consider incorporating more detailed information on specific diseases, this study provides valuable scientific evidence regarding illness perceptions in diverse linguistic populations. It also suggests research directions for exploring the association between language assistance and illness perception among non-Japanese-speaking patients.
